# Apple Vision Pro-Enabled Endoscopic Endonasal Approach for Pituitary Adenoma Resection: First In-Human Spatial Computing Skull Base Neurosurgery Case Report

**DOI:** 10.1055/a-2945-8171

**Published:** 2026-10-06

**Authors:** Regan M. Shanahan, Ethan Crosby, Griffin Hurt, Shovan Bhatia, Brock Gjesdal, Michael R. Kann, Prajeet Santra, Calvin Brinkman, Rishi Basdeo, Garret W. Choby, Georgios A. Zenonos, Jacob T. Biehl, Edward G. Andrews

**Affiliations:** 1 6614 University of Pittsburgh School of MedicinePittsburghPennsylvaniaUnited States; 2 Department of Neurological Surgery 6595 University of Pittsburgh Medical CenterPittsburghPennsylvaniaUnited States; 3 6614 School of Computing and Information, University of PittsburghPittsburghPennsylvaniaUnited States; 4 Department of Mechanical Engineering 6612 Carnegie Mellon UniversityPittsburghPennsylvaniaUnited States; 5 Department of Otolaryngology–Head and Neck Surgery 6595 University of Pittsburgh Medical CenterPittsburghPennsylvaniaUnited States

**Keywords:** skull base, Apple Vision Pro, endoscopic endonasal approach, augmented reality, spatial computing, case report

## Abstract

**Objective** This study aimed to report the first in-human skull base neurosurgery case using Apple Vision Pro (AVP).

**Main Outcome Measures** Endoscopic stream with the AVP in an endoscopic endonasal transsellar and left transcavernous approach for resection of a pituitary adenoma.

**Results** Endoscopic endonasal approach was performed using the AVP for resection of a pituitary adenoma. The procedure was completed without complication. A postoperative magnetic resonance imaging (MRI) showed no convincing residual pituitary mass. The patient was discharged on postoperative day 2.

**Conclusion** Endoscopic streaming to the AVP is safe in skull base surgery. We described the first case report of an AVP-specific spatial computing technology successfully used to display endoscopic feed to the skull base neurosurgeon during skull base neurosurgery with no intraoperative complications. Continued integration of intraoperative navigation platforms, patient vitals, preoperative imaging, and electronic medical records with endoscopic streams serves as an important step in optimizing surgeon experience and developing a virtual operating room.

## Introduction

Spatial computing is increasingly used in surgery to overlay digital information directly into the surgeon’s field of view, creating a blend of real anatomy and virtual data. By integrating imaging, navigation, and electronic medical records into a head-mounted display, spatial computing can reduce the need to look away from the operative field, potentially improving ergonomics and situational awareness. Secondary benefits of spatial computing include remote collaboration and enhanced teaching capabilities. In spatial computing-augmented spine surgery, preoperative planning and simulation have been coupled with intraoperative navigation and visualization to improve pedicle screw and interbody device placement. 1 2 3 More recently, the Apple Vision Pro (AVP) has successfully been used in endoscopic spine surgery. 4

Spatial computing has recently entered the field of skull base surgery, offering new ways to integrate digital information into the surgeon’s field of view during complex endoscopic procedures. 5 6 The endoscopic endonasal approach (EEA) for pituitary tumors requires surgeons to rely on high-definition video and real-time navigation to safely work within narrow anatomical corridors adjacent to critical neurovascular structures. 7 Preliminary studies evaluating the benefits of spatial computing streaming and tumor overlays discuss potential for reduction in violation of critical structures, optimized exposure of the sella, early identification of neurovascular structures, and improved training and education. 6 Head-mounted displays now allow simultaneous visualization of the endoscopic feed alongside key perioperative data, such as navigation images and patient information, without requiring the surgeon to look away from the operative field or conform to the space limitations set by the external monitors and operative setup.

This emerging workflow has the potential to improve ergonomics, streamline access to essential imaging and clinical data, and enhance intraoperative surgeon experience during EEA pituitary surgery. Here, we present a case report describing the real-time display of the surgical video feed to the surgeon on the AVP during endoscopic endonasal resection of pituitary tumor. We discuss the feasibility and perceived benefits of this technology in skull base neurosurgery.

## Illustrative Case

This study was approved by the University of Pittsburgh Institutional Review Board (STUDY25070057). Written informed consent was obtained from the patient to participate in the study before the day of surgery.

### Patient History

The patient is a 38-year-old female with a past medical history of hypertension, type 2 diabetes mellitus, and Cushing disease who presented to neurosurgery clinic for management of a pituitary tumor. She presented with insomnia and excessive weight gain. Physical exam was notable for hirsutism, moon facies, and abdominal striae. The patient was neurologically intact with 20/40 vision in both eyes. Confirmatory laboratory workup included elevated adrenocorticotropic hormone (ACTH), salivary cortisol, 24-hour free urine cortisol, abnormal dexamethasone suppression test, and abnormal desmopressin stimulation.

Contrasted magnetic resonance imaging (MRI) revealed a 15-mm mass filling the sella turcica consistent with a functional pituitary adenoma given concurrent laboratory findings. There was suspected involvement of the medial aspect of the left cavernous sinus. Otherwise, no suprasellar extension or pressure on the optic chiasm ( Fig. 1 ).

**Fig. 1:**
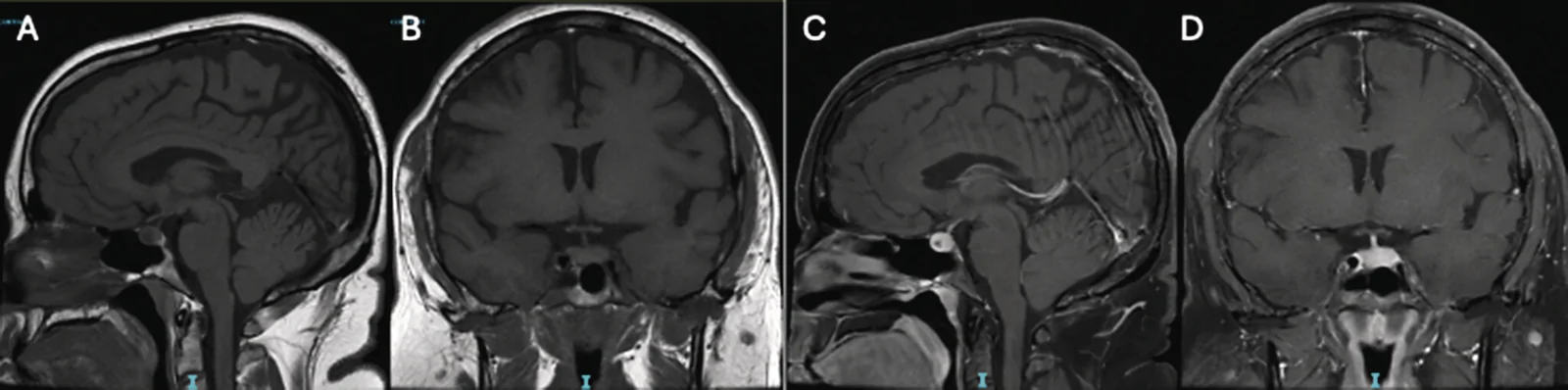
Preoperative T1 precontrast ( **A** ) sagittal and ( **B** ) coronal, and postcontrast ( **C** ) sagittal and ( **D** ) coronal magnetic resonance images demonstrate a 15-mm pituitary adenoma.

### Description of Apple Vision Pro

The AVP consists of 5 sensors, 6 microphones, and 12 external cameras to record and project the external environment into the headset display. Each eye display has a resolution of 3660 × 3200, providing high-quality video and data to the user wearing the headset, supporting 90, 96, and 100 Hz refresh rates. Battery tests were conducted before the case by streaming the endoscopic video feed until the battery system was depleted; the stock AVP battery lasted roughly 90 min under operating conditions. With an aftermarket backup battery installed, the battery system was shown to last for 3 h and 15 min during testing. In the event that the surgery exceeded planned battery time, the designed system allowed for sterile “hot swapping” of the backup batteries without the need to reboot the AVP or have the surgeon leave the sterile field.

### Surgeon’s Familiarity with Apple Vision Pro

Before the AVP was used in the operative setting, the surgeon was trained to ensure he was comfortable with the system. Headset fit was determined using Apple’s Vision Pro Fit software to measure the user’s face, and the need for corrective lenses was determined by an independent contemporary optometry evaluation. The surgeon was cued on proper donning of the headset to ensure proper fit, comfort, and maintain sterility throughout the duration of the case. The surgeon practiced opening and operating the software that displays the endoscopic stream in the operating room. The surgeon was briefed on the exit procedure should he need to remove the headset during the surgery, which consisted of the surgeon removing the AVP and battery with assistance of the circulating nurse and/or member of the research team.

### Intraoperative Hardware Setup

To facilitate AVP use within this case, the research team established a connection between the endoscopic video feed and the headset. The high-definition endoscopic video was connected by a Serial Digital Interface (SDI) cable from the endoscopic camera control unit in the operating room to specialized capture hardware. The SDI cable allowed transmission of ultrahigh-definition digital video signals. The function of the hardware and its software was to convert raw video signals into a compressed and containerized format, suitable for network transmission. The connection between the AVP and the specialized hardware was mediated by a private local access network (LAN) encrypted using the Wi-Fi Protected Access 3 (WPA3) protocol. This prevented any devices outside of the surgeon’s designated headset from viewing the endoscopic feed. The network’s connected device list, available from the Wi-Fi router’s control panel, was also monitored for any unknown devices as an additional precaution against unauthorized viewing. The video data delivered from the hardware were ephemeral; no patient information or images were retained on the specialized hardware nor the AVP. All data were handled following institutional protocols as approved by the University of Pittsburgh Institutional Review Board (STUDY25070057). The hardware was positioned near the endoscopic camera control unit, away from the operative field, and was connected to a wireless network shared with the AVP.

The endoscopic feed was recorded by a wired capture card, which was connected to the DisplayPort output of the endoscope control unit using a DisplayPort to high-definition multimedia interface (HDMI) cable. The capture card recorded onto a 256-GB SD card for the entirety of the case. A view of the operating room operative field was recorded using a Blackmagic Studio Camera 4K Pro, which was equipped with a 4-TB solid-state drive. The setup for the endoscopic streaming and recording devices took 64 min to complete.

### Surgery

The patient was prepped and draped in standard fashion. Otolaryngology colleagues began the procedure and harvested a right-sided nasoseptal flap using standard protocol. 8 9

### Apple Vision Pro Intraoperative Setup

Before the AVP was provided to the neurosurgeon, the front and inside lens covers were removed, and the corrective lens inserts (ZEISS Optical) were secured to the inside lens. To maintain sterility, the surgeon donned the AVP prior to scrubbing. After powering on the device, the surgeon calibrated the eye tracking and interpupillary distance using standard approaches in the Apple software. Next, the surgeon initiated the streaming software, confirming appropriate latency, brightness, and color fidelity. Following setup, the surgeon scrubbed while wearing the headset. Once scrubbed, the surgeon anchored the virtual display according to preference. With the AVP, the surgeon was still able to see all pertinent objects in the operating room, including instrument cables, the patient, surgical assistant, and conventional monitors through the pass-through video without any clinically meaningful distortion noted by the surgeon. Although the software allows the video feed to change angle automatically to follow the surgeon’s movements while remaining fixed in position, the surgeon did not elect to use this feature during the case. A two-stream configuration incorporating Medtronic StealthStation navigation ( Fig. 2 ) was available; however, for study purposes, only the endoscopic stream was displayed ( Fig. 3 ). The otolaryngology team relied on the standard operating room monitor display throughout the case.

**Fig. 2:**
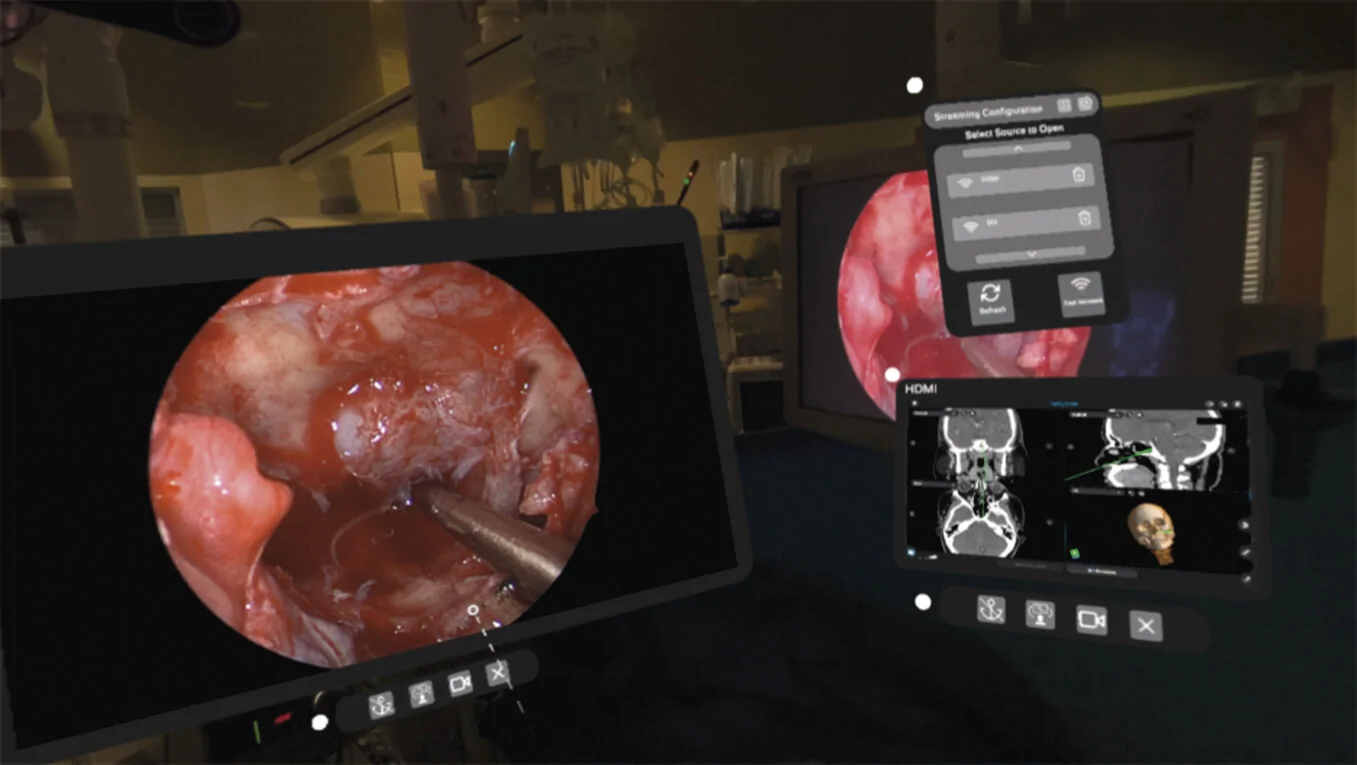
Intraoperative view from AVP displaying endoscopic video feed (left) and Medtronic StealthStation navigation platform (lower right). AVP, Apple Vision Pro.

**Fig. 3:**
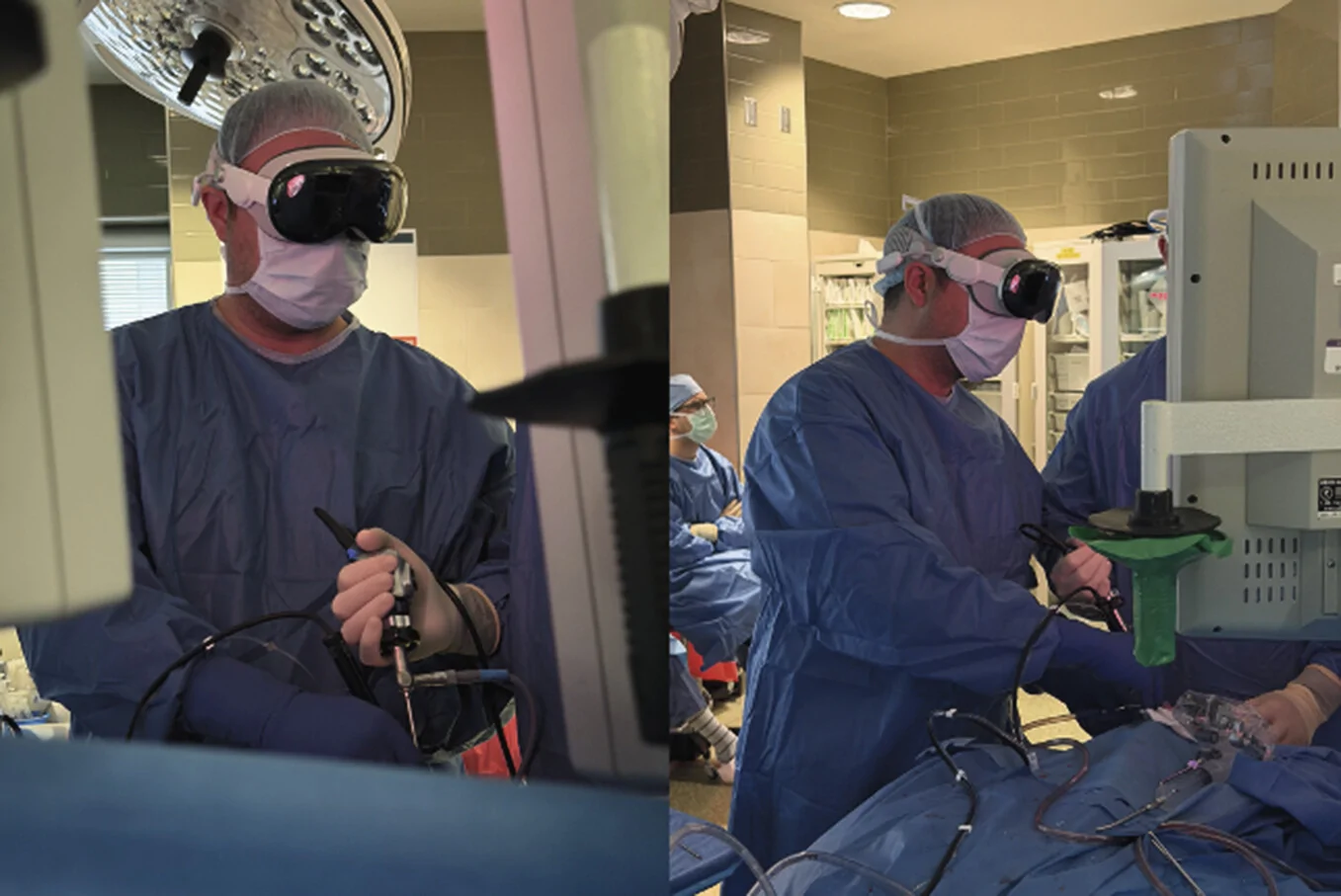
Neurosurgeon performing the operation while visualizing the endoscopic video in mixed reality.

The attending neurosurgeon began with bony drilling of the sellar bone, thinning it and then completing removal with a Kerrison rongeur. Bilateral parasellar drilling exposed the internal carotid arteries and cavernous sinuses. After dural opening, tumor resection proceeded. Intraoperatively, the tumor was noted to abut and traverse the medial wall of the left cavernous sinus. The surgical team therefore elected to proceed with a left transcavernous approach, including dissection of the medial cavernous sinus wall. An incision was made in the periosteal dura, and Surgifoam was injected into the cavernous sinus. A feather blade was used to incise dura along the carotid artery, and the medial wall was carefully dissected. The inferior hypophyseal artery was identified, coagulated, and divided. The carotico-clinoidal ligament was similarly identified and meticulously dissected free from the carotid artery. Dissection continued posteriorly along the carotid artery until the medial wall was fully mobilized, enabling complete tumor resection. No intraoperative cerebrospinal fluid leak was observed. The otolaryngology team then resumed reconstruction, successfully placing the nasoseptal flap. The patient was admitted to the intensive care unit (ICU) for close neuromonitoring.

Throughout the procedure, the endoscopic video stream was transmitted to the AVP without perceptible delay, interruption, or system failure. Upon completion of the case, the surgeon removed the AVP and battery pack, then scrubbed out. The surgeon was administered a Simulator Sickness Questionnaire (SSQ) 10 and Surgery Task Load Index (SURG-TLX). 11 SSQ (16 symptoms on a 4-point scale [0–3]) is a validated tool used to quantify symptoms such as nausea, oculomotor strain, and disorientation associated with immersive technologies, with higher scores indicating greater symptom severity. SURG-TLX (six domains on a Likert scale) is a validated measure of perceived surgical workload across domains, including mental demand, physical demand, temporal demand, task complexity, situational stress, and distractions, with higher scores reflecting greater workload. On the SSQ, the surgeon scored 13.92 in the category of disorientation, 0 in the category of nausea, and 22.74 in the oculomotor category, for a total score of 11.22. Due to the time constraints of clinical practice, the surgeon completed only the Likert component of the SURG-TLX on the day of surgery. The surgeon scored a 26 out of a possible 120 on the unweighted SURG-TLX.

Postoperative MRI demonstrated expected postoperative changes without convincing residual pituitary mass ( Fig. 4 ). The patient had no postoperative deficits and was discharged from the ICU on postoperative day 1 with discharge from the hospital on postoperative day 2. Cortisol levels appropriately declined during admission. At outpatient follow-up on postoperative day 5, the patient demonstrated appropriate healing with no evidence of cerebrospinal fluid leak.

**Fig. 4:**
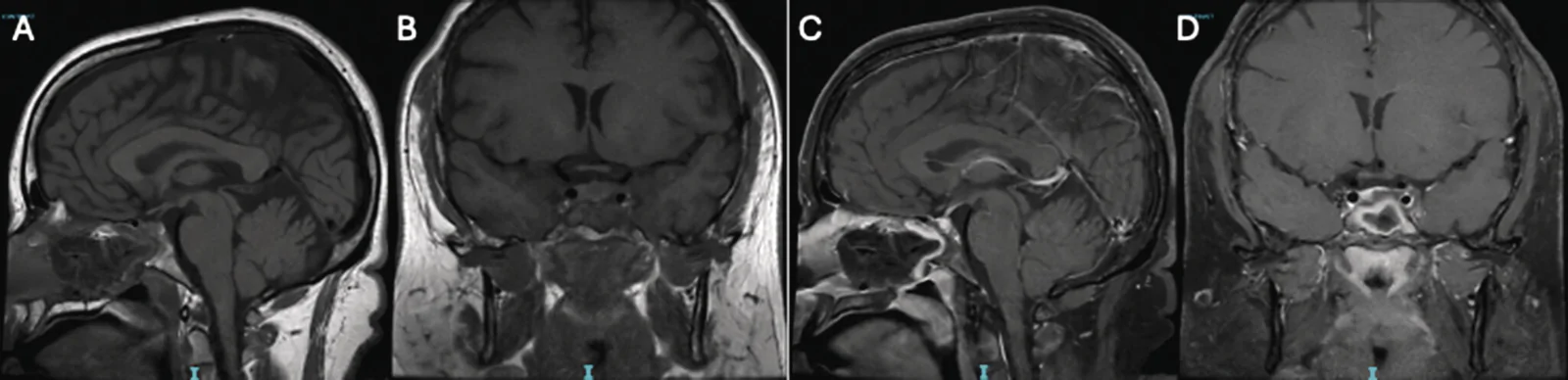
Postoperative T1 precontrast ( **A** ) sagittal and ( **B** ) coronal, and postcontrast ( **C** ) sagittal and ( **D** ) coronal magnetic resonance imaging demonstrate no residual pituitary mass.

## Discussion

EEA surgery combines complex, high-risk anatomy and depends heavily on intraoperative technology. This makes it a strong use case for spatial computing platforms aimed at improving surgical performance. 6 This case represents the first in-human use of the AVP as a spatial computing headset to display a live endoscopic stream during pituitary adenoma resection. The attending skull base surgeon completed the entire operation using the AVP feed without complication or workflow disruption. Consistent with prior neurosurgical spatial computing experience, this case demonstrates that real-time, head-mounted visualization of the operative field is technically feasible, ergonomically acceptable, and compatible with contemporary skull base team dynamics. 12 13

### Early Spatial Computing Skull Base Studies

This first in-human streaming case with an AVP builds on prior cadaveric and intraoperative studies. In a recent study from Choi et al, skull base neurosurgeons performed four cadaveric endonasal skull base dissections, comparing standard two-dimensional planar displays with mixed-reality streaming interfaces. 14 On an objective error analysis, surgical performance was comparable between the two conditions. Moreover, there was not a discernible difference in participants’ ability to distinguish between mixed-reality streaming and two-dimensional planar display conditions. In separate intraoperative studies, Zhang et al and Goto et al demonstrated that three-dimensional computer graphic models superimposed on the video monitor during endoscopic transsphenoidal surgery can enhance, and in some cases outperform, traditional displays. 5 15 Taken together, these early investigations suggest that spatial computing has expansive potential both as a primary visualization stream and as a supplementary tool to improve navigation to support intraoperative decision-making.

### Feasibility and Technical Performance

In this case, the AVP was integrated into the existing EEA workflow by pulling the live endoscopic video from the endoscope camera control unit via SDI into dedicated hardware for real-time wireless streaming to the headset. System setup occurred alongside routine preoperative preparation. The setup did not interfere with intraoperative neuromonitoring, and there was no evidence of degraded or altered neurophysiological signals. Hardware and software performance were stable throughout the case. The endoscopic stream displayed on the AVP was comparable in quality to the standard monitor. The surgeon completed all aspects of the neurosurgical portion of the case, including delicate carotid dissection and resection of the medial cavernous wall, without difficulty or error. On postoperative survey, the surgeon commented that the virtual display enhanced task focus when compared with the traditional monitor configuration. Common side effects of spatial computing include “simulator sickness” and symptoms of fatigue, nausea, vomiting, and the possibility of headaches and eye strain from the device. 16 With continued exposure to the device in an intraoperative setting, these symptoms should be minimized as the surgeon continues to calibrate to the technology over time.

### Ergonomics

Adoption of the AVP in endoscopic transsphenoidal surgery may improve ergonomics and workflow in the operating room. In the confined operative and monitoring fields where neurosurgeons and otolaryngologists work simultaneously, the AVP offers flexibility by eliminating dependence on fixed monitor locations. Individualized displays unique to each AVP user are advantageous for visualization of critical anatomy. The virtual screen is larger and adjustable to surgeon preference. AVP displays can be adjusted to match each surgeon’s height and visual preferences to optimize the line of sight.

### Limitations

Our single-patient case report is limited by lack of a control group, subjective assessment, short follow-up, and single-surgeon experience with the technology. Practical constraints include headset weight, battery life, intraoperative usability, and dependence on stable network connectivity. From an enablement perspective, however, the streaming pipeline implemented here is best viewed as a minimal “first feed” that establishes an extensible pathway for delivering a broader intraoperative information landscape to a head-mounted display, including concurrent video sources (endoscope, neuronavigation, microscopy, fluoroscopy), physiologic monitoring, imaging review, and context-aware overlays. This framing shifts the headset from a remote display of a single camera feed to a surgeon-facing dashboard that can unify disparate operating room signals into one consistent, glanceable interface. Scaling beyond a single feed motivates future work on stream orchestration and synchronization, bandwidth and quality-of-service management, multistream user interface (UI) composition (layout, prioritization, rapid switching), and explicit fallback behavior when systems degrade that are aligned with surgical phase and task demands.

The technology is also limited by possible motion sickness and eye strain in surgeons, particularly during prolonged use or when head motion, display refresh, or stream jitter produces a mismatch between visual motion cues and vestibular sensation. Eye strain may be exacerbated by fixed focal depth, vergence–accommodation conflict, imperfect fit or interpupillary alignment, and sustained attention to high-contrast content in the peripheral visual field. These factors motivate incremental acclimation and training, the necessity for planned and careful device fit, and interface design choices that minimize unnecessary motion and visual clutter.

### Future Directions

A larger series will be completed at our institution to continue to assess safety, quantitative ergonomic and performance metrics, and user experience. Integration in the operating room will be an iterative process to fully integrate into the complex, multidisciplinary landscape of the operating room. Future technical integrations that unify additional intraoperative data sources and enable intelligent, context-aware alerts during critical moments will continue to expand the utility of this innovation in the operating room, both in neurosurgery and across other procedural domains.

## Conclusion

Spatial computing technology continues to permeate the surgical landscape with great success across multiple surgical disciplines. We described the first case report of an AVP-specific spatial computing technology successfully used to display endoscopic feed to the skull base neurosurgeon during an EEA surgery for pituitary adenoma resection. In this single case, AVP use was technically feasible and did not require deviation from the standard surgical workflow. As spatial computing hardware and software continue to evolve, such platforms may transition from simple video display to a more comprehensive intraoperative workspace to enhance surgeon ergonomics, visualization, and intraoperative decision-making in skull base neurosurgery.
